# Farmers’ Perceptions on Implementing Automatic Milking Systems in Large USA Dairies: Decision-Making Process, Management Practices, Labor, and Herd Performance

**DOI:** 10.3390/ani14020218

**Published:** 2024-01-09

**Authors:** Camila Flavia de Assis Lage, Thaisa Campos Marques, Daniela R. Bruno, Marcia I. Endres, Fernanda Ferreira, Ana Paula Alves Pires, Karen Leão, Fabio Soares de Lima

**Affiliations:** 1 Cornell Cooperative Extension, Cornell University, Bath, ME 14810, USA; cd546@cornell.edu; 2 Department of Population Health and Reproduction, University of California, Davis, CA 95616, USA; tcmarques@ucdavis.edu (T.C.M.); nandadobbins@gmail.com (F.F.); 3 Departmento de Zootecnia, Instituto Federal Goiano, Rio Verde 75901-970, GO, Brazil; piresanapaulazoo@gmail.com (A.P.A.P.); karen.leao@ifgoiano.edu.br (K.L.); 4 Cooperative Extension, University of California Agriculture and Natural Resources, Fresno, CA 93701, USA; dfbruno@ucanr.edu; 5 Department of Animal Science, University of Minnesota, Saint Paul, MN 55108, USA; miendres@umn.edu

**Keywords:** robotic milking, adoption, cow health, cow welfare

## Abstract

**Simple Summary:**

Automatic milking box systems are becoming more common in USA dairy farms despite being costlier than traditional methods. This study investigated farmers’ perceptions on why farms with at least seven milking boxes opted for them, focusing on technological changes, farm structure, management, labor, cow health, and milk production. The primary reasons for choosing these systems were labor cost reduction, improved cow welfare, and enhanced milk production. Most farms constructed new barns with open stalls and easy cow movement to facilitate use of these systems. Farmers perceived that the adoption led to higher water and energy usage, while the operational costs remained manageable. Farmers reported labor savings, better working conditions, and more effective management of cow health issues like mastitis and foot problems. The impact on milk production and quality was mixed, with some farms experiencing improvements. Similarly, farmers perceived an improvement in reproductive performance, supported by activity data. Overall, the shift to Automatic Milking Systems was viewed positively by more than half of farmers, who recommended it to others, although they advised careful consideration of specific farm needs and conditions. These are initial experiences rather than conclusive evidence.

**Abstract:**

Automatic Milking System (AMS) installations are increasing in the USA despite the higher investment cost than conventional systems. Surveys on AMSs conducted outside the USA focused on small–medium herds, specific regions, or aspects of AMS milking. This study described farmers’ perceptions about the decision-making process of adopting an AMS in the USA’s large dairies (≥7 AMS boxes) regarding changes in technology, housing, management practices, labor, herd performance, and health. After being contacted, 27 of 55 farmers from large AMS herds completed the survey. The main reasons for adopting an AMS were labor costs, cows’ welfare, and herd performance. Most farms constructed new barns, used a free-flow traffic system, and changed their feed management. Increases in water and energy use were perceived by 42% and 62% of farmers, respectively. Farmers estimated decreases in labor costs of over 21%, and AMS employees worked 40–60 h/week. Milk production increases were reported by 58%, with 32% observing higher milk fat and protein content. Easier sick cow detection, better mastitis management, and improvements in pregnancy rates were reported. Thus, farmers transitioning to AMSs perceived altered resource utilization, labor cost savings, and improvements in employee quality of life, animal welfare, and farm management. While 54% of respondents would recommend an AMS to other farms, 38% suggested considering additional aspects prior to adoption.

## 1. Introduction

Automated Milking Systems (AMSs) have become popular in Europe, Canada, and New Zealand in the last 30 years [1,2,3]. However, the adoption and implementation vary based on regional practices and dairy farming characteristics. In Europe, Canada, and New Zealand, AMSs are commonly used in smaller- to medium-sized dairy herds (e.g., 80 cows on average), mainly in confinement housing [1,4]. Contrastingly, in New Zealand and Australia, AMSs are often employed in larger dairy herds (e.g., 390 cows), usually in pasture-based farms [5].

The situation in the USA is quite distinct from these regions regarding dairy farming demographics, trends, and practices. In addition, the number of American dairy herds has decreased from 600,000 in the 80s to ~30,000, whereas the average herd size increased from 74 to 337 cows [6]. Thus, this transition to larger dairy herds and various challenges related to the sustainability of the dairy sector, such as fluctuating milk prices, high feed costs, and limited margins for return on investment, might be some of the factors slowing the adoption of AMSs in the USA compared to Canada, Europe, and Oceania.

Recently, comprehensive AMS surveys were carried out in Canada and Australia, underscoring aspects such as housing, farm management, cow health, cow training, transition challenges, human–animal relationship, quality of life, labor management, and milk performance [4,5,7,8]. Albeit these surveys were very informative, they included local small herds (median of 77 to 85 lactating cows) or pasture-based systems that do not necessarily represent the USA demographic considering the adoption of an AMS. A limited number of surveys performed in the USA focused on a few specific topics, such as milk quality [9], milking aspects [9,10,11], transitioning concerns [12], or adoption in specific states [13,14]. Piwczynski et al. [15] highlighted that the average number of robots per herd in the USA is higher than in certain European countries (2.71 vs. 1.88). Therefore, many of the perceptions from previous surveys might not fully represent the demographics or directly apply to those large dairy operations in the USA adopting AMSs.

**Figure 1:**
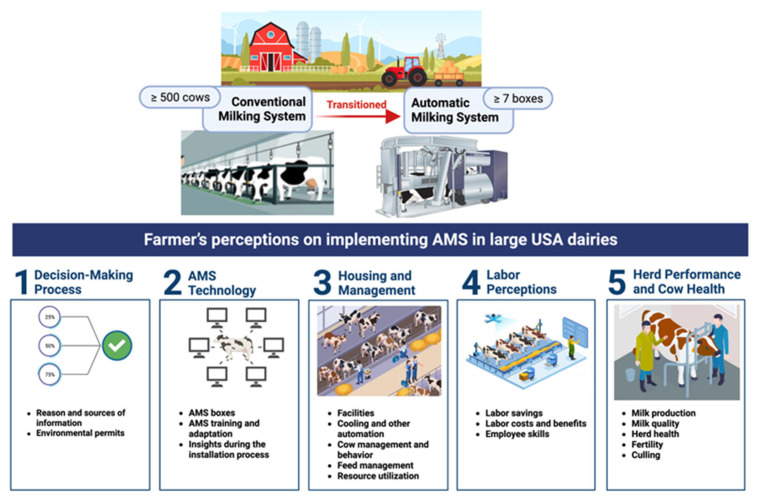
Study design and objectives of the Automatic Milking System (AMS) survey.

The global milking robot market is experiencing significant growth, estimated to reach USD 4.31 billion by 2027, with 7.2% coming from the USA [16] and larger herds potentially considering transitioning to an AMS. However, the literature has limited information on the challenges and perceptions of large dairy herd producers in the USA transitioning to an AMS. Therefore, this study was designed to specifically assess AMS adoption in large dairy herds within the USA framework and potentially highlight emerging challenges and trends in the dairy sector. This focus on large farms in the USA is vital, as they face unique challenges in adopting AMSs, a trend increasingly relevant due to their larger herd sizes and higher number of robots per farm compared to other regions. Our objectives were to explore perceptions and experiences of large dairy farms in the USA (≥7 AMS boxes) that transitioned to an AMS regarding the decision-making process, AMS technology, housing and management practices, labor, herd performance, and cow health (Figure 1).

## 2. Materials and Methods

The study protocol was reviewed and approved by the University of California, Davis Institutional Review Board (IRB ID: 1685201-1).

### 2.1. Farm Selection and Survey Design

To establish the cut-off point for the USA’s large dairies, we used USDA’s classification of farms with ≥500 lactating cows [17]. However, AMS farms have constraints on the number of cows that can be milked in one unit, varying from 55 to 70 cows/box. In that way, using the cut-off point of 500 lactating cows would limit the number of farms that had already adopted this technology when the survey was applied. Based on field research and personal communication with consultants and stakeholders, the threshold of ≥7 AMS boxes installed (minimum of 385 to 490 lactating cows) would allow us to represent the large dairies transitioning to AMSs for this study.

A questionnaire was developed (File 1, https://figshare.com/articles/dataset/AMS_in_Large_Herds_UC_Davis_Survey_pdf/23915847 (accessed on 12 November 2023)) based on [4] containing 164 questions categorized into five sections: decision-making process; AMS technology; housing and management practices; labor; herd performance and cow health. The questionnaire was initially tested with two farmers from California, who confirmed that it covered all the aspects that farmers in the region were interested in learning more about.

The question format included a single choice of multiple options, multiple choices of multiple options, and a fill-in-the-blank option when the choice “other” was selected (a blank space was added to be filled by the respondent). The number of respondents per question varied depending on which survey question was presented and because respondents were given the option to answer or skip any questions. In addition, because respondents could choose more than one answer for some questions, percentages can sum higher than 100% for some of the questions.

For this survey, a convenience sample [18] of 55 farms across the USA was obtained through Lely USA (Pella, IA, USA) and DeLaval USA (Vernon Hills, IL, USA), extension agents from different land grant universities, consultants, veterinarians, and lenders, among others. The available literature does not provide specific information on the number of farms in the USA that have transitioned to an AMS using ≥7 AMS boxes. However, according to personal communication with AMS dealers, we estimated that approximately 100 farms within the threshold of ≥7 AMS boxes were operational in the USA when the survey was performed.

All producers in our sampling frame were contacted, and data were only obtained from those who agreed to participate. The participating farms were contacted by mail, phone, email, and in person from March 2021 to the end of December 2021. All surveys were completed through an online version of the study created on Qualtrics^®^ (Qualtrics Research Suite, Provo, UT, USA). Respondents were allowed to stop the survey at any point, in which case those surveys were excluded.

### 2.2. Statistical Analysis

Due to the exploratory nature of the project, the data analyses primarily included descriptive statistics (means, SD, medians, 1st–3rd quartiles, percentages) performed with the MEANS and FREQ procedures of SAS 9.4.

## 3. Results

### 3.1. Demographic Characteristics

Our survey was completed by 27 out of 55 AMS producers (49% response rate), while 3 respondents opened the questionnaire but never answered. The respondents were from 13 states: California (*n* = 3), Indiana (*n* = 2), Iowa (*n* = 1), Kansas (*n* = 1), Michigan (*n* = 1), Minnesota (*n* = 2), New Hampshire (*n* = 1), New York (*n* = 6), Pennsylvania (*n* = 1), Texas (*n* = 2), Vermont (*n* = 1), Washington (*n* = 1), and Wisconsin (*n* = 5). The range of time since transitioning to AMS was 1.1 to 16.3 yr, with a median of 4.4 yr when the survey was applied.

The information presented was provided by part owners (56%), full owners (22%), and herd managers (22%). Thirty-seven percent of respondents were <35 years old, thirty percent were between 36 and 45 years old, twenty-two percent were between 46 and 56, and seven percent were over 56 years old. One respondent left this question blank (4%).

### 3.2. Herd Characteristics

Herd characteristics and robot variables are presented in Table 1. Most of the herds (63%) consisted entirely of purebreds (94% Holstein and 6% Jersey). In the remaining herds (37%), the percentage of Holstein ranged from 33 to 99%, followed by Jersey (1–33%) and crossbreds (1–100%).

Among respondents (*n* = 27), 52% plan on having or already have their entire herd under an AMS, 37% do not, and 11% do not know. Regarding future growth plans, of the 26 respondents, 54% plan to expand their herd, while the remaining 46% do not.

### 3.3. Decision-Making Process for Adopting AMS

#### 3.3.1. Reason and Sources of Information

Reducing labor costs and improving cow’s welfare and herd performance were the main reasons for adopting an AMS (Table 2).

The primary sources of information farmers consulted included the AMS dealer, visiting farmers already using an AMS, and technical magazines (Table 3).

Technical assistance during the decision-making process and availability of technical support after AMS installation were the most common reasons respondents chose a particular AMS brand, as detailed in Table 4.

#### 3.3.2. Environmental Permits

Just 22% of respondents had to apply for specific environmental permits due to transitioning to an AMS, which did not include reasons directly related to the technology, but rather reasons related to herd expansion or specific to certain regions (i.e., nutrient management plan updates due to changes in manure management or water use, increase on the number of animals).

### 3.4. AMS Technology

#### 3.4.1. AMS Boxes

Out of the 27 respondents, 26 installed new AMS and one installed both new and used AMS boxes. Robot brands were Lely (59%) and DeLaval (41%). Among the respondents (*n* = 26), they believe the lifespan of their AMS to be 10–15 years (39%), 15–20 years (38%), >20 years (15%), and <10 years (8%). Regarding resale, 56% did not foresee selling their used AMS during or after its lifespan, while 44% considered potential resale. A significant variation was observed in the expected salvage value of AMS boxes (respondents, n = 24), with 21% believing this value could be 20–30% of the original price (Table 5).

When asked if they replaced or thought about replacing their AMS for newer versions (respondents, *n* = 26), 38% intended to use their boxes until their lifespan ends (i.e., end of warranty or making a beneficial upgrade), 35% would consider a replacement if the productivity increases are worth the extra costs, 31% are ready to replace theirs (stopped working or unusable), and 15% are actively planning a replacement and have talked to their dealer about it.

#### 3.4.2. AMS Training and Adaptation

Out of 26 respondents, no training was given to 85% of the employees before transitioning to an AMS, 11% received training from AMS dealers, and 4% answered that employees received training from an independent consultant. Conversely, the employees’ adaptation to the new management system after transitioning to an AMS was extremely or somewhat easy (62%), easy (19%), or difficult (19%). Moreover, just 35% had to hire new employees with different skills after transitioning, while 65% answered that they did not.

Regarding farmers’ adaptation to the changes in management and automation of their farm due to the AMS (respondents, *n* = 27), 93% were either significantly or very adapted, 4% moderately adapted, and 4% slightly adapted. In the same way, when asked about their employees (respondents, *n* = 25), 76% were either significantly or very adapted, 20% were moderately adapted, and 4% were slightly adapted.

#### 3.4.3. Insights during the Installation Process

During the AMS installation process (respondents, *n* = 25), 68% would do something different, including modifications to barn design (32%), cow flow (16%), training of employees before transitioning (24%), cow selection on AMS startup (8%), and 8% other. Table 6 shows the main information they would like to have received before AMS installation.

### 3.5. Housing and Management Practices

#### 3.5.1. Facilities

Out of 27 respondents, 19 built new barns (70%), whereas 19% retrofitted existing facilities, and 11% retrofitted and built new barns. Just two farms (7%) changed the housing system from dry lots to free stalls during the transition from conventional milking systems (CMSs) to AMSs, since all farms manage cows under free-stall systems. The construction cost per stall (excluding the AMS cost) varied among respondents (Table 7). Free-flow traffic systems were used by 69%, followed by guided-flow (19%) and both (12%) on the same farm.

When the survey was applied, 58% still used their conventional parlor after installing an AMS (respondents, *n* = 26). The main reasons for keeping the conventional parlor (respondents, *n* = 15) were that the farm was still transitioning to the AMS and did not have enough robots for the whole herd (73%), to milk hospital and fresh cows (60%), and “other” reasons (27%) that included “We only milked with a parlor for a few weeks then it was too much work and upkeep to keep using it”; “Cows that would not adapt to AMS, it is not used”; “It just has not been removed”; and “Low producers and late lactation cows”.

#### 3.5.2. Cooling and Other Automation

Panel or basket fans (44%) and soaker systems (37%) were the most common cooling strategies, followed by cross-ventilation (30%), tunnel ventilation (22%), big ceiling fans (7%), misting systems (7%), and positive pressure ventilation (4%). Almost half (48%) had more than one cooling system for lactating cows. All farms had fans over the beds, the AMS waiting area (85%), and the feed bunk (77%). Almost all (85%) also cooled dry cows with shade, fans, and/or soakers.

Other types of automation were present in 81% of the dairies, such as automatic feed pushers (74%), cow brushes (67%), robotic manure scrappers (15%), and automated milk feeders and training feeders (15%). The automated scraper was more common (67%) than flush systems (30%) to clean the alleys of barns on AMS farms. Recycled manure solids (52%) and sand (34%) were the main types of bedding used (respondents, n = 23).

#### 3.5.3. Cow Management and Behavior

The main criteria for selecting cows to be transferred from CMSs to AMSs (respondents, n = 15) was udder conformation (73%), followed by cow behavior (27%), milking speed (20%), only high-producing cows (13%), random (4%), and 27% other (age and all cows).

Sixty-seven percent did not provide any training for heifers and cows prior to milking them in an AMS. Heifers spent 4–7 days (62%) adapting to robots, whereas cows ranged between 4–7 days (38%) and 8–10 days (21%). Most of the farms (93%) managed lactating cows in two or more groups, 67% had a separate pen for first lactation cows, and 41% housed fresh cows and hospital cows separately.

Sixty-four percent (respondents, *n* = 25) did not follow the manufacturer’s recommendation regarding the number of cows per robot due to being focused on milk harvested per robot (forty-four percent), over the maximum capacity (twenty-five percent), and under the maximum capacity (thirty-one percent). Most of the farms (85%) mentioned between 2.5 and 3 milking visits per robot (respondents, *n* = 27), with 91% spending 6 to 8 min per visit (respondents, *n* = 23). Almost half (48%) of farms fetched under five cows per day per robot, 30% between six and ten cows, and 22% more than eleven cows. Regarding the waiting period before fetching cows (respondents, *n* = 26), 73% reported 7–11 h, and 23% waited 12–16 h for early lactation up 100 DIM cows. In the case of mid- to late-lactation cows, the protocol was slightly different, with 65% reporting a wait time of 12–16 h, and 23% reported waiting 7–11 h.

Of 26 respondents, 62% perceived an improvement in cow comfort after transitioning to an AMS. Almost all respondents (93%) reported that they perceived cows as calmer, and 52% said cows seemed to spend more time lying down.

#### 3.5.4. Feed Management

After transitioning to an AMS, 85% changed feed management, and 81% provided a partial mixed ration. The number of times feed was delivered stayed the same in 70% of farms, while 19% increased and 4% decreased. The daily number of feedings was mainly once (48%) and twice (41%), while a minority reported thrice (4%) and more than three (7%). Of 25 respondents, 96% pushed up feed more than three times/d and had headlocks in the whole barn (56%) or only in sorting pens (40%).

#### 3.5.5. Resource Utilization

Regarding resource utilization (*n* = 24), 42% perceived increased water consumption, and 30% mentioned that it seemed unchanged. Meanwhile, 62% estimated increased energy consumption, 27% that it stayed the same, and 11% perceived that it decreased. For the monthly maintenance cost/AMS box (excluding cleaning and disinfecting products), 50% answered that it costs < USD 1000, 29% between USD 1000 and 1500, 8% between USD 1500 and 2000, and 13% over USD 2000.

### 3.6. Labor Perceptions

#### 3.6.1. Labor Savings

**Figure 2:**
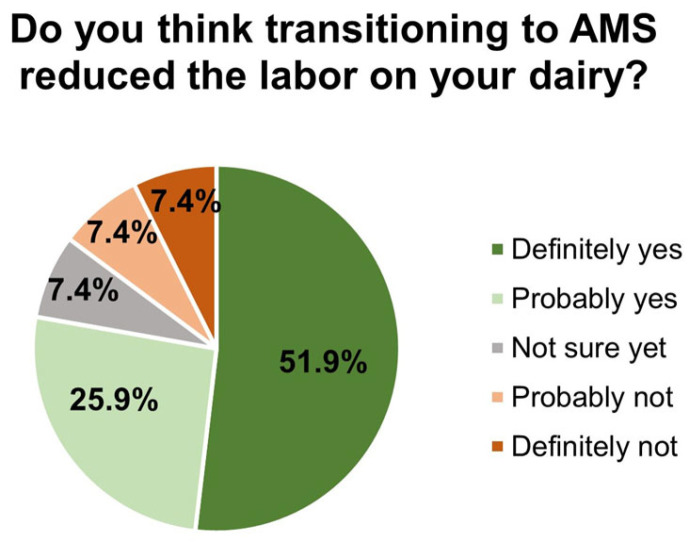
Do you think transitioning to automatic milking systems reduced the labor on your dairy? (27 respondents chose ≥ 1 reason).

Farmers perceived a reduction in labor at the farm after transitioning to an AMS (Figure 2), decreasing the full-time employees by 30–50% (22%), 10–20% (17%), 20–30% (13%), and >50% (13%). Interestingly, of 26 respondents, 35% needed to hire more employees with different skills after transitioning to an AMS.

On average, full-time employees worked (*n* = 26) between 40 and 60 h/wk (77%), >60 h (19%), and <40 h (4%). Forty-six percent estimated that employees spent 2–5 h/d fetching cows, followed by <2 h (twenty-seven percent), 6–10 h (twenty-three percent), and >11 h (four percent).

#### 3.6.2. Labor Costs and Benefits

Seventy-four percent perceived a reduction in labor costs since transitioning to an AMS (respondents, *n* = 23). The breakdown of the decrease was >21% (35%), 10–20% (30%), and <10% (9%). In contrast, 17% reported no change in labor costs, and 9% noticed an increase.

Comparing employees who work directly with the robots to other employees at the farm (respondents, n = 25), 80% had no increased wages, and 20% reported additional benefits such as increased wages and improved quality of life. Additionally, the AMS employees’ hourly wage received was over USD 15 (54%), USD 13–15 (38%), and USD 10–12 (8%).

#### 3.6.3. Employee Skills

When asked which skills are desired in employees to work with AMSs, 78% of the respondents (*n* = 21) answered that the essential skills are “a calmer and respectful person who knows how to deal with cows and is worried about their welfare” and “that they must be willing to change their mindset regarding managing cows”. In addition, 74% of the respondents (*n* = 20) indicated that employees must have good initiative and the ability to make decisions and be interested in learning about the new system and software, and 7% of the respondents (*n* = 2) answered “other”, including self-motivation and mechanical ability to fix robots.

### 3.7. Herd Performance and Cow Health

#### 3.7.1. Milk Production and Milk Quality

**Figure 3:**
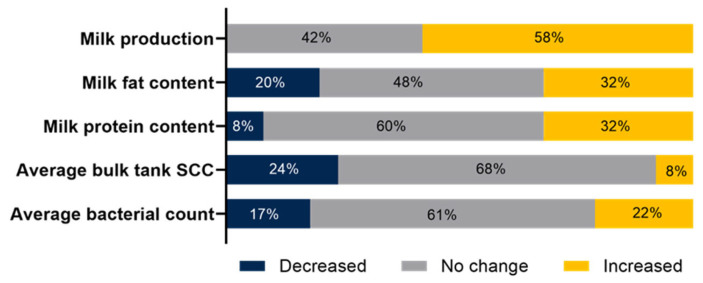
Milk performance after transitioning to Automatic Milking System. SCC = somatic cell count.

Milk performance after transitioning to an Automatic Milking System is represented in Figure 3.

#### 3.7.2. Herd Health

**Figure 4:**
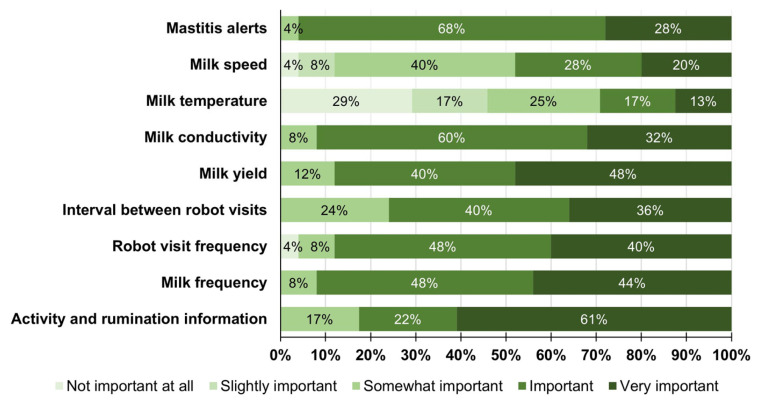
How important do you think the data generated by the AMS systems are for your herd health management?

After transitioning to an AMS, 88% perceived that detecting sick cows became easier. The importance of the data generated by the AMSs for herd health management according to the farmers is presented in Figure 4. Most farms (85%) utilized activity and rumination data, primarily for breeding cows (100%) but also for disease detection (87%). Regarding the overall transition in cow health, almost half (56%) perceived that it stayed the same, 37% that it had improved, and 7% that it was impaired.

The clinical mastitis rate was perceived as not changed by 44%, and a decrease was perceived by 33%. Improvements in mastitis management were estimated by 58% of farmers, with increases in mastitis detection perceived to be most likely due to the robots’ features of identifying cows with mammary gland inflammation. Alerts for mastitis were used by 59% to check all cows, and 48% combined the alert with information from activity or other sensors before checking on the cow. Of the respondents who treated their cows with antibiotics after the mastitis alert (*n* = 9), 67% either checked their cows first for clinical signs of disease, or 33% visually checked them and cultured their milk. Of 24 respondents, the use of teat sealant during the dry period was perceived to have increased from 79% to 96% after transitioning to an AMS.

Sixty percent of the respondents (*n* = 25) perceived an improvement in lameness detection, sixty-two percent perceived decreases in lameness prevalence (respondents, *n* = 26), ninety-five percent adopted hoof trimming (respondents, *n* = 19), and eighty-five percent implemented foot bathing after switching to an AMS (23 of 27). Respondents (*n* = 24) believed that the displaced abomasum rate was unaltered (54%), decreased (33%), and increased (21%).

#### 3.7.3. Fertility and Culling

**Figure 5:**
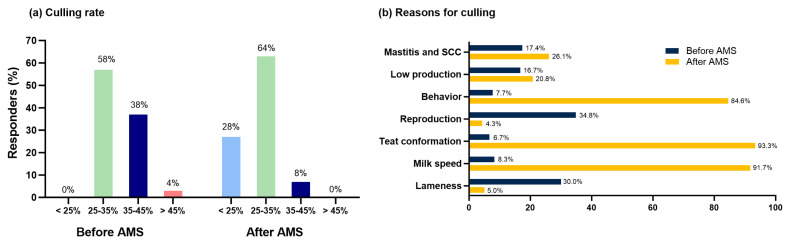
Culling rates (**a**) and main reasons for culling cows before and after transitioning to AMS (**b**). SCC = somatic cell count.

The respondents (*n* = 25) estimated that the pregnancy rate increased (60%), stayed the same (36%), or decreased (4%). Although 59% of respondents indicated that they had the same reproductive programs, 33% of the respondents indicated that they were relying more on activity data to breed cows. Moreover, one-third of the dairies surveyed used genomic testing to improve traits related to AMS efficiency. Figure 5 shows the culling rates (Figure 5a) and the main reasons for culling cows before and after transitioning to an AMS (Figure 5b).

### 3.8. Statement of Expectations

Overall, 89% of all respondents were extremely or somewhat satisfied with the support provided by the AMS dealer, whereas 11% felt neutral or somewhat dissatisfied. Additionally, 46% reported they could obtain immediate assistance from their dealers within 1 h of the request, and 54% within 3 h.

**Figure 6:**
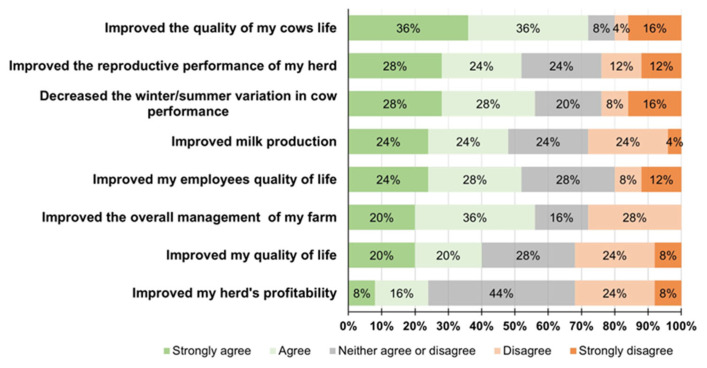
Farmers’ perceptions about transitioning to Automatic Milking Systems on a Likert Scale (27 respondents chose ≥ 1 reason).

Respondents’ perceptions of transitioning to an AMS on a Likert Scale are shown in Figure 6. Out of 25 respondents, more than half strongly agree or agree that the AMS has improved the quality of cows’ lives and their reproductive performance, decreased winter/summer variation in milk production, and improved overall management of the farm and employees’ quality of life. On the other hand, less than 50% strongly agree or agree that the AMS improved milk production, farmers’ quality of life, and herd profitability.

**Figure 7:**
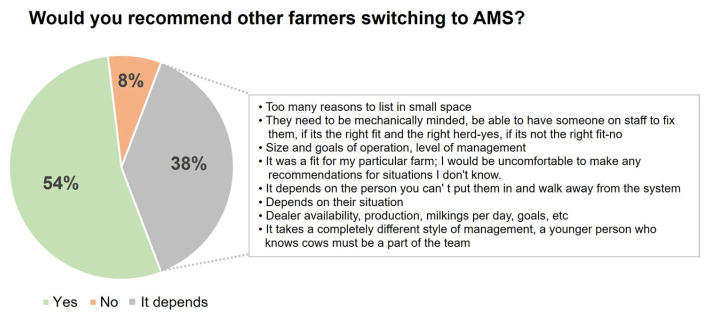
Would you recommend that other farmers switch to Automatic Milking Systems? (27 respondents chose ≥ 1 reason). The reasons for those who chose “It depends” are pointed out in the rectangle.

The farmers’ recommendation to their peers regarding the transition to Automated Milking Systems (AMSs) is shown in Figure 7.

## 4. Discussion

Our study collected information about the perceptions of a sample of large dairy farmers in the USA who transitioned to an AMS. This includes insights into their decision-making process, AMS technology adopted, housing and management practices implemented, labor differences, herd performance, and cow health. These insights help to describe the landscape of large dairy herds adopting AMSs in the USA. However, it is crucial to emphasize that these are preliminary perceptions. To establish a more comprehensive and scientifically robust understanding, it is essential to verify or falsify these perceptions through systematic analysis of herd data. This follow-up study would provide empirical evidence to support or challenge the initial observations and experiences reported by the farmers, thereby contributing to a more nuanced and data-driven understanding of the impact of AMSs on large dairy farms.

Labor savings consistently emerged as the primary reason for adopting AMSs across global regions. Surveys from Europe and Canada additionally highlighted the aim to enhance dairy farming family lifestyles and quality of life [7,11,19,20], which was not an aspect indicated by the respondents in the current survey. Indeed, our survey revealed that even other aspects, such as cow welfare and herd performance, were perceived as more important reasons for AMS adoption. These differing top reasons can be likely attributed to each region’s farm structures and priorities. Canadian and European farms tend to be smaller and depend more on family labor. The respondents representing large USA herds adopting AMSs suggest a culture of this particular community that tends to list herd productivity and efficiency over their quality of life. Another potential explanation for the differences is the fact that the respondents in large dairy herds represent managers and part owners who do not perceive quality of life as a reason that aligns with their role.

Most producers relied on information from AMS suppliers and farmers familiar with AMSs, which aligns with findings from European and Canadian surveys [7,21,22] underscoring that independent of the size of dairy herds and the particular characteristics of a region, AMS adopters rely on the AMS community as a main source to learn and decide about the adoption of the technology.

The present study also observed varied perceptions about AMS lifespan, salvage value, and plans for replacing boxes. The expected lifespan range was between 10 and 20 years, which is aligned with the manufacturer’s estimated lifespan (~13 years) [23,24]. Our survey found that a small proportion of respondents do not see salvage value for their boxes and expect to use them until the end of their lifespan without a plan for replacing them. Steeneveld et al. [25] reported that other than higher capital costs, the use of an AMS rather than a CMS does not affect farm efficiency, but reliable estimates of AMS lifespan remain scarce. Therefore, future studies that rigorously assess AMS lifespan are needed to elucidate more accurate realistic expectations and overall AMS costs.

In line with previous studies [3,4], producers often preferred to construct new facilities for AMSs instead of retrofitting existing barns. These preferences persist even though our study reported costs were higher than upgrading current barns. Regarding changes in housing systems, most Canadian farms (47%) moved from tie stalls to free stalls, and in the present study, just 7% of farms changed from dry lots to free stalls. The housing shift being less prominent in the USA could be due to a major proportion of dairies already using free stalls, as highlighted in a mid-Atlantic region survey by [13]. Furthermore, a study by [14] in Idaho found that farms with free stalls were 60% more likely to adopt an AMS compared to those with different systems. A significant portion of farms kept the old parlor (58%) alongside the AMS, including farms that had completely switched the entire herd to an AMS. The primary reasons were to milk hospital and fresh cows, or those that did not adapt to the AMS. In addition, 37% did not plan to milk all their cows in the AMS, suggesting a continued use of both systems. Further studies are needed to understand why some farmers resist the full transition to AMSs, which would help identify the associated risks and opportunities in large herds.

Our survey found that the median number of cows per box in USA dairy farms (60 ± 3 cows/box) was higher than [4] reported in a Canadian study (51 ± 9 cows/box). In our survey, 15% of farms operated over maximum capacity, and 27% focused on milk harvested per robot, which may account for the differences in the cows-per-box ratio. This result aligns with [26]’s findings, which highlighted that a crucial part of the system’s profitability depends on the milk harvested by the robot each day, which in turn depends on the number of cows milked and the frequency of milkings per cow [27]. On the other hand, comparing the times when the surveys were conducted, these differences can also be related to technological advances and a better understanding of system management for improved milk efficiency.

Regarding resource utilization, increases in water and energy consumption were perceived by 42% and 62% of the farms, respectively. Recently, a study was conducted in Germany [28] to evaluate the impact of different technologies on the energy efficiency of AMSs. An AMS equipped with an electrical drive of the attachment arm showed strong advantages in the energetic efficiency of the whole milking process. However, the scarcity of research on AMS resource utilization and the impact on sustainability need to be further investigated, which was pointed out in a recent scoping review of our group [29].

Consistent with previous surveys [7,19,22], most farmers perceived a reduction in labor, with a large portion estimating decreases of more than 21%. Similarly, previous studies reported reductions of 18–19% [30,31] or over 29% [32], while others found no change in labor use between CMS and AMS farms [25,33]. The variation could be attributed to individual farmer management skills, such as profitability or labor savings with AMS [34].

In agreement with European and Canadian surveys [7,35], most farms implemented the free-flow traffic system. In the current study, cow welfare ranked as the second reason to adopt AMSs, followed by the possibility of enhancing herd performance. In the context of increasing farm intensification, public concern over animal welfare has grown, as noted by [36]. An Australian study by [5] found that transitioning from CMSs to AMSs led to farmers interacting less with their cows. However, cows in AMSs exhibited less fear around humans, showing shorter avoidance distances and reduced stress during close handling. Thus, adopting technologies like AMSs appears to enhance cow welfare, such as by minimizing milking wait times, which is a significant concern for many large dairy farmers.

After adopting AMSs, many respondents noticed a rise in milk production (58%), with slight changes in milk components (32%) and unchanged levels of SCC and bacterial counts. The Canadian survey [8] reported even more producers (82%) observing increased milk yields without changes in milk quality. However, it is noteworthy that misconceptions about perceived increased milk production have been reported previously. A recent Canadian survey [37] showed that ~60% of dairy producers either misconceived or were unaware of the actual changes in their milk yield and SCC. Moreover, recent Canadian studies [38,39] associated milk production and quality in AMSs with herd-level housing factors and management practices rather than the AMS alone. Therefore, it is reasonable to surmise that future studies more rigorously isolating the relative impact of AMS adoption on milk production and quality are needed for large dairy herds representing the USA demographics captured by the current survey.

Similar to [4], most respondents perceived that the AMS’s data made detecting sick cows easier, with clinical mastitis rates remaining stable or decreasing. Analogous to the Canadian survey, the current study revealed that the perception of lameness detection improved in most herds after AMS adoption, and the perceived incidence of lameness decreased. The similarity in trends observed by the survey suggests that AMSs, independent of their size, seem to facilitate routine assessment of cows that are behaving differently (e.g., cows not making their way to robots, lower activity, lower rumination, altered milk conductivity) or underperforming (e. g., decreased milk production).

Regarding pregnancy rates, as indicated by [4], producers estimated improved reproductive performance and relied more on activity to breed cows. As discussed above, for perceptions regarding milk production, a robust assessment isolating the impact of AMS adoption from changes in reproductive programs is needed to represent an AMS’s impact on fertility outcomes fully.

In our study, respondents believed the pattern of the culling rate changed after transitioning to an AMS, with an increase from 0% to 28% of respondents estimating a culling rate lower than 25%. These findings are not aligned with the previous survey, which indicated that perceptions of culling rates remained unchanged [4]. An explanation for the potential differences is inaccuracies in the perceptions of our respondents or simply differences in genetics and behavior that allowed lower culling due to the udder conformation and adaptation to robots than reported in studies performed in Canada and Europe. As shown previously, our respondents indicated that behavior and udder conformation have overtaken reproduction as the primary reasons for culling cows [4]. Research has shown that factors such as cow behavior and udder conformation can compromise the overall success of the AMS, with it being more economically viable to remove the problematic cows from the herd [40]. Cow behaviors that consistently disrupt the system or require excessive manual intervention may influence the availability, frequency of milkings, and efficiency of the AMS [40]. Cows with poor udder conformation posed challenges for teat cup attachment and were fetched twice as often as those without conformation problems [40].

In general, most of the respondents had a positive perception of transitioning to an AMS and would recommend it to other farmers. This finding aligns with previous studies [4,31] that found that the AMS improves the quality of life for cows and employees and overall farm management. This finding agrees with [7] that the quality of life for owners improved to a lesser extent on large AMS farms. The authors speculated that producers with larger herds would not score the statement “AMS has improved the quality of my life” as positively compared to smaller farm producers because they would not be able to reduce the number of employees to the same extent as smaller farms, which is corroborated by the findings of the present survey. On the other hand, in our study, we need to consider that one-third of respondents did not agree with the statements that AMS improved milk production, farmers’ quality of life, and herd profitability. This underscores the necessity for a more nuanced understanding of AMS adoption, considering individual farm contexts and broader socio-economic factors.

Finally, an improvement in herds’ profitability was not perceived by most of the respondents. The findings align with variable results reporting the estimated costs of AMSs. Higher initial and maintenance costs of AMSs in previous simulations and observational studies have suggested AMSs to be less profitable than CMSs [13,23]. However, [41] found that larger AMS farms operating for over four years generate a higher gross farm income than newer and smaller ones. In our survey, the median number of years since AMS adoption was 4.4 years, but there were farms with adoption just over one year, which could have potentially influenced the profitability perceptions. In addition, advancements in AMS technology suggest that AMSs could be more profitable under certain circumstances such as increasing wages, higher milk production, and a longer equipment life span [23,42,43]. As forecasted by these studies and accelerated by the COVID-19 pandemic, increased labor wages and labor shortages are already a reality for most farmers. Many studies like [24] created simulation models on economic performance in different scenarios as tools for farmers to decide on AMS investment. However, updates in these models, including updated wages and inflation, need to be implemented to predict the risks of adopting AMSs more accurately.

Lastly, as in most surveys, the potential exists for misinterpretation of questions and recall bias (an issue of remembering accurately). In addition, [4] mentioned that farmers who transitioned to AMSs might be affected by the cognitive justification in which a purchaser of an expensive product looks past any product faults to justify their purchase [44]. Nonetheless, this survey focused on the perceptions of large dairy herd respondents transitioning to AMSs. The data presented here may benefit farmers thinking about transitioning to AMSs, future researchers seeking research directions, and extension educators/outreach personnel focusing on educational programs. 

## 5. Conclusions

Overall, our study showed that regarding large dairy herds transitioning to AMSs, respondents perceived improved milk performance, cow welfare, and labor savings. Moreover, they would recommend the technology to other farmers but emphasized that success depends on farm aspects, farmer expectations/mindset, and dealer proximity/relationship. Considering that several factors affect profitability when the farm implements an AMS, the economic aspect of the investment needs to be further investigated to help AMS adopters have a better overview of the AMS. Updated simulation models for economic evaluations, including updated wages, inflation, and milk production scenarios, are needed to help farmers accurately predict the risks and opportunities of adopting AMSs. Lastly, the insights garnered from this study not only provide a foundational reference for future research but also offer guidance to large dairy farmers considering the switch to AMSs, enabling them to make more informed decisions that best align with their operational goals.

## Figures and Tables

**Table 1:** General herd characteristics and robot variables of respondent herds on the AMS survey (*n* = 27).

Herd Characteristics	*n*	Average	SD	Median	Min	Max	p25	p75
Lactating cows	27	2150	3235	940	405	17,000	535	2250
Lactating cows in AMS	27	819	437	720	400	2200	475	1074
% cows on AMS/herd	27	69	30	83	4	100	44	100
Number of AMS boxes	23	14.2	7.0	12	7	36	8	18
Number of cows/boxes	22	60	3	60	51	65	59	62
Years since adoption	27	5.7	4	4.4	1.1	16.3	2.5	7.4

**Table 2:** The main reasons respondents decided to transition to an Automatic Milking System (AMS) (27 respondents could choose ≥ 1 reason).

Main Reasons for Transitioning to AMS	Respondents	
	*n*	%
Reduce labor cost	22	81
Improve cows’ welfare	21	78
Improve herd performance	20	74
Reduce the number of employees at the farm	19	70
Increase the technology of my farm	18	67
Improve my quality of life	12	44
Other *	4	15

* “Maximizing the genetic potential of my cows and having more accurate data to market the genetics of bulls born from my cows”; “Reliability over hired help”; “The quality of life is a hard one when you are the only one that now how to make it run”; “Updating the farm to get the best technology available”.

**Table 3:** The main sources of information farmers consulted before adopting Automatic Milking Systems (AMSs) (27 respondents could choose ≥ 1 reason).

Sources of Information Consulted before Adopting AMS	Respondents	
	*n*	%
Supplier of AMS (manufacturer and dealer)	27	100
I visited farms that had installed AMS	26	96
Technical dairy magazines and newsletters	18	67
Online information (blogs, Youtube videos, etc)	11	41
Nutritionists and other consultants	10	37
Local/regional meetings (University or AMS companies)	7	26
Veterinarian	4	15
Research papers or university extension fact sheets	4	15
On-farm demonstration and workshops	3	11
Podcasts	1	4
Webinars	0	0

**Table 4:** Reasons respondents choose an Automatic Milking System (AMS) brand when transitioning from conventional to AMSs (27 respondents could choose ≥ 1 reason).

Sources of Information Consulted before Adopting AMS	Respondents	
	*n*	%
Availability of technical support after installation of AMS	20	74
Technical assistance during the decision-making process	20	74
Personal communication with manufacturer/dealer	14	52
Personal preference for a specific brand design and functionalities	14	52
Price (cost of installation and maintenance)	10	37
Technological package associated with the AMS	10	37
Veterinarian, nutritionist, or other technical recommendation	6	22
Other *	6	22
Financing options	1	4

* “Believed it was the best product to maximize cow performance and comfort”; “Excellent dealership support and close dealership proximity”; “Preferred sleek robotic arm”; “Reliability”; “Robustness of equipment”; “Support team was better, and technology has superior quality in my opinion”; “These units are quiet”.

**Table 5:** How much will the automatic milking system box’s salvage value (residual value) be after the expected lifespan (*n* = 24)?

Salvage Value of the Box after the Expected Lifespan	Respondents	
	*n*	%
<5%	1	4
5–10%	4	17
10–20%	4	17
20–30%	5	21
30–40%	0	0
40–50%	4	17
>50%	1	4
I do not think I will be able to sell my old robot	5	21

**Table 6:** Which additional piece of information or training would you like to have received before automatic milking systems (AMS)? (25 respondents could choose ≥ 1 reason).

An Additional Piece of Information before Installation of the AMS	Respondents	
	*n*	%
AMS routine maintenance	13	50
AMS software training	12	46
Specific employee training on AMS	11	42
How to train cows on AMS	10	39
I had all information I needed at the time	9	35
Feed quality and management	6	23
Other *	4	15

* “Too many for this small space to list”; “Listen less to the dealer and more to people that work directly with robots and cows on other farms”. Changes in the barn included “A bigger sorting area”; “Footbaths (are tough no matter what)”; “I would employ a different design on the retrofit barns to make it a more modified-guided flow”; “Add sort pens in some groups”; “Double wide foot bath”; “I would have more water trough”; “Knowing what we know now, I would employ a different design on the retrofit barns to make it a more modified-guided flow”; “Increase the number of robots”; “Ventilation system”; “Minor details”.

**Table 7:** Construction costs (USD/stall, excluding the AMS cost) in herds that built or retrofitted barns (*n* = 26).

Cost of Construction/Stall	New (*n* = 20)	Retrofit (*n* = 6)
<USD 1500/stall	3 (15%)	4 (67%)
between USD 1500 and 2500/stall	3 (15%)	1 (16.5%)
between USD 2500 and 3500/stall	4 (20%)	1 (16.5%)
between USD 3500 and 5000/stall	4 (20%)	0
>USD 5000/stall	6 (30%)	0

## Data Availability

Data are contained within the article.
